# Enteral nutrition on intensive care unit: a protocol based approach

**DOI:** 10.1186/2197-425X-3-S1-A180

**Published:** 2015-10-01

**Authors:** G Jesus, SM Fernandes, A Alvarez, J Gouveia, H Bento, C França

**Affiliations:** Centro Hospitalar Lisboa Norte, Serviço de Medicina Intensiva, Lisbon, Portugal; Faculdade de Medicina da Universidade de Lisboa, Lisbon, Portugal; Faculdade de Medicina da Universidade de Lisboa, Disciplina de Medicina Intensiva, Lisbon, Portugal

## Introduction

Underfeeding is still reported as a major therapeutic failure in intensive care units (ICU), despite the increasing recognition of nutritional support as a predictor of patients' outcome. Early optimized enteral nutrition is reported to diminish infectious events, length of stay (LoS) and, with less preponderance. Implementation of nutritional protocols is likely to improve enteral feeding in ICUs^1^.

## Objectives

We studied nutrition support adequacy after implementation of an enteral nutritional protocol in a university hospital ICU.

## Methods

We implemented a nutritional protocol based on American and European guidelines recommendations. The protocol consisted on daily adjustment of enteral support using a excel based tool designed by the study authors aiming to accomplish 100% of caloric and proteic requirements. We compared nutritional support during the first 5 days of ICU stay in all admitted patients pre and post protocol implementation, excluding patients with indication for parenteral nutrition. Primary objective was the difference of caloric and proteic intake between the two periods. Secondary objectives were possible side-effects of early protocolled nutrition, LoS, mortality, days of invasive ventilation and development of pneumonia. Comparison between groups was performed using parametric and non-parametric tests as adequate.

## Results

We included 70 ICU admissions, 35 pre-protocol and 35 after protocol implementation, with similar distribution of severity criteria as well as reason for ICU admission. We observed a significant increase in caloric intake after the fifth day of stay (79% versus 100% of proposed caloric intake, p < 0.001) and of proteic intake since the first day (30% versus 45% of proposed proteic intake administered, p < 0.001) after protocol implementation. Importantly, at day 5 of ICU stay the average protein intake was 80% after protocol and 48% before protocol (p < 0.001). There were no differences between groups on the average length of stay, survival, incidence of pneumonia or days of mechanical ventilation. We also did not observe increased frequency of adverse events like increased gastric aspirates, urea or diarrhea.

## Discussion

This pilot study shows the feasibility and efficacy of a simple nutritional protocol, which resulted in improvement of nutritional support without increasing expectable adverse effects. Similar simple design protocols might be widely applied, leading to an increase in nutritional support to ICU patients and hopefully to improved mortality and decreased number of ICU related complications like motor disability.
